# An overview of blockchain science and engineering

**DOI:** 10.1098/rsos.200168

**Published:** 2020-06-03

**Authors:** Ghassan Karame, Michael Huth, Claire Vishik

**Affiliations:** 1 NEC Laboratories Europe, Heidelberg, Germany; 2 Imperial College London, London, UK; 3 Intel, Austin, TX, USA

**Keywords:** cryptography, distributed systems, networks, trusted computing

## Abstract

This is the preface to a special issue in the journal Royal Society Open Science, themed around blockchain technology. Since this is still an emergent and interdisciplinary field, we first provide a gentle introduction into that larger topic. Then, we discuss why this technology has been criticized for not being energy-efficient. Next, we provide an analysis of recent developments in blockchain research that may help with making blockchain technology truly sustainable. Finally, we highlight some of the contributions made by papers in this special issue.

## 1. Introduction

We are pleased to present this special issue of a collection of research papers on blockchain technology (BCT) and distributed ledger technology (DLT). These are vibrant research areas with high impact, notably in their applications to cryptocurrencies such as Bitcoin and Facebook’s recently introduced Libra. But this technology can also be used to create resilient trust anchors in open systems, for example, for the veracity and provenance of critical information such as the mileage of a car that is to be resold.

This foreword is not the place for a genuine introduction into these topics, we refer instead to [1,2] as sources for more in-depth introductions. But let us at least offer some working definitions here. DLT is used to achieve consensus about the replication of data or state machines across a geographically distributed network and where the consensus and its management typically do not rely on a central administrator. A state machine is a device that stores a status and that updates this status and may perform other actions, both based on received input.

Blockchains may be seen as instances of such DLT solutions where the data and its change history are presented in a linear chain of blocks that are cryptographically linked to make them resilient against unintentional or malicious manipulation.

DLT solutions may also use graph-based structures rather than linear chains, for example as in IOTA’s Tangle [3] or Hedera’s Hashgraph [4], and this can offer advantages such as better scalability of transactions volumes. Unfortunately, terminology is not yet established in this space—which standardization initiatives such as the ISO/TC 307 [5] will help with addressing: the terms ‘blockchain’ and ‘distributed ledger’ are often used interchangeably or there can be confusion about their meaning. Innovation departments, at present, may see DLT projects as a PR exercise and often lack a deeper, in-house understanding of this technology in order to transfer use cases into production. Such lack of knowledge can also lead to ill-informed decisions when choosing instances of such technology and supporting project partners.

There is little doubt that BCT/DLT has brought a lot of innovation, mostly in its combination of tools from cryptography, distributed systems and programming languages. This was powerfully demonstrated in the creation and launch of Bitcoin, which allows anyone to join this network to trade in the digital currency ‘bitcoin.’ Trust into this network is a self-emergent property that results from the interplay of several factors, one of them being the monetary incentivization of miners—parties that specialize in solving a cryptographic puzzle called Proof of Work.

The initial excitement around BCT/DLT was probably hyped up by some, yet its technical offerings become more mature now, their innovations are here to stay, and they will make their way into many products and infrastructures. Still, several research challenges remain for this technology, some of which are addressed in this special themed issue. Let us mention the need for making DLT systems resilient to attacks based on quantum computing and the requirement for more scalable information processing.

On the latter point, the first formative phase of BCT and DLT research and development was apparently conducted by people in applied cryptography, distributed systems, networking, and—to some extent—programming languages. There seems to have been little involvement in that phase by people from the areas of database systems and information retrieval, for example, in the design and implementation of the blockchain framework Hyperledger Fabric [6].

It is thus not too surprising that the so-called third-generation blockchains, including Algorand [7], are now aiming to solve problems many of which have been familiar to the database research community for a rather long time. For example, the sharding of chains—that is hoped to help with the scalability of transaction processing throughput on blockchains—is related to the topic of denormalization of databases. It seems that there is great potential in bringing the database, information retrieval, and BCT/DLT communities closer together so that they can share problems and solutions more effectively in the years to come. The recent Dagstuhl Seminar *Distributed Computing with Permissioned Blockchains and Databases* [8] appears to have been a good step in that direction.

Let us also comment on the considerable hype around initial coin offerings (ICOs), which use smart contracts on an existing blockchain to operationalize the offering of a token as an investment into a new project—typically the development of a BCT/DLT system. A smart contract is a deterministic program supported through a blockchain. The integrity of a smart contract can be verified by anyone on the blockchain. The execution of a smart contract is deterministic, trackable and irreversible in as much as the underlying blockchain offers those qualities.

In some major financial markets, more money seems to have been invested in ICOs than in conventional initial public offerings (IPOs) in 2017. But 2018 saw a decline in the volume of investment for ICOs, in part due to the regulatory uncertainty around the legal status of tokens as a financial instrument, and also because some blockchain projects appeared to operate a ‘pump and dump’ scheme.

Principles and best practice from business ethics should thus inform the operation and assessment of blockchain projects. We refer to [9] for a survey and framework on that important topic. These principles ought to also guide any approaches to deciding whether a blockchain would be subject to a software update that brakes the immutability of the chain, e.g. by rewinding the chain to a point in the past and invalidating all transactions that happened since. In [10], an ethical framework informed by a Kantian view is proposed that can help with deciding whether enacting such so-called hard forks would be ethical.

On the regulatory side, we now see more clarity in that space in many territories. Some countries—including Switzerland, Singapore and Malta—are now actively fostering the development of financial instruments based on BCT/DLT technology and its cryptocurrencies. Facebook’s Libra may be seen as a related and strong strategic signal by a major ICT company in that space; we refer to [11] for a discussion and review of that project. We are likely to see similar token use in production in Internet of things (IoT) and mobility, for example with digital platforms for car sharing.

## 2. Blockchain and sustainability

Proof of Work is at the heart of the resiliency of the Bitcoin system: each new block added to the chain is the result of a leadership race in which miners compete against each other by attempting to solve a hard cryptographic puzzle. For the latter, a miner combines part of the current blockchain state, new transactions that should be included in the chain, and some random source into an input for a hash function. The puzzle is solved by varying the value of the random input part until the hash of the combined input has a certain minimal number of leading 0 bits. The value of this parameter is adjusted periodically and, over time, has increased dramatically—reflecting the competition and reward structure of this mining process and advances in hardware manufacturing that further fuelled such competition. On 3 January 2010, this value was 1.183 and this increased several orders of magnitude to 5.6186 × 10^12^ only 9 years later. As a consequence, if Bitcoin were a country, it would now consume more energy than Chile, Venezuela and The Philippines. Bitcoin supports the trustworthy processing and recording of less than a dozen transactions per second (tps), some estimate this to be as low as 5 tps. Therefore, the energy demands of Bitcoin seem to be extraordinary and extremely wasteful. In fact, this huge demand of energy would appear to not be ecologically sustainable even if the system were able to support tps rates such as those occurring for credit card companies and their global transaction processing: Visa does not require as much energy as Chile.

These concerns have, therefore, motivated research into designing BCT/DLT systems that have a much lower energy footprint than Bitcoin. Byzantine fault-tolerant consensus protocols, such as the one used in the Hyperledger Fabric [12], offer considerable advantages here as they do not require the solving of energy-hungry puzzles but achieve consensus through the staged and stateful communication of messages. The higher communication complexity of such protocols, however, means that in practice there is a bound on the number of nodes that can participate in this consensus process. In certain use cases, this may be unacceptable since such a number of nodes (for example, less than 20) would then have to be trusted as an ‘oligarchy’ with the faithful management of the system.

Algorand’s consensus protocol, in contrast, is aiming to combine the strengths of both approaches for synergistic benefits:

B1 Random choices provide for strong system security and resiliency: for Bitcoin this is the random nature of the mining race.
B2 Non-random consensus protocols are much more energy-efficient: for Byzantine protocols, consensus is computed with much lower energy consumption.

Algorand harmonizes the seemingly conflicting benefits B1 and B2 by retaining the small size of nodes that participate in consensus creation but by randomly selecting that set of nodes anew for each step of that consensus computation. This random selection is achieved by means of a publicly verifiable random function. This function is a sequence of seeds where the genesis block contains the initial seed and the seed of the next block is determined by the seed of the last block and the digital signature of the leader who produces the new block. Therefore, this approach appears to retain system security and resiliency (which Bitcoin achieved only with great energy consumption) and creates consensus within energy budgets of normal ICT processing.

Algorand is not the only blockchain that seems to reduce the energy demands for consensus, both IOTA and Hashgraph seem to have similar advantages. One may see such efforts as important contributions to the sustainability aspect in security and privacy research and development. More generally, it seems important to develop design principles for systems that will optimally trade-off energy consumption and carbon footprint with a desired level of trustworthiness of system services (for example, for ‘consensus’ as a system service).

While we think that such research is vital in order to make our increasingly digitized worlds more ecologically sustainable, it is worth pointing out that the sustainability of digital technology should not be seen in isolation of system components, services or consumer products. Of course, it is useful to understand the energy demands of the direct system usage and to try and contain such demands at design, implementation or operation stages. ‘Energy’ is here understood broadly, to include the efforts required in the production or transformation of materials, products or infrastructure.

Indeed, much of the existing literature that researched energy demand has focused on the direct consumption of consumer products, such as television sets, smart-phones and so forth. However, it seems equally important to understand the energy needs of infrastructures. For example, the lower power consumption of handheld devices compared to desktop computers seems to make consumption more sustainable. But the increased connectedness of these devices has certainly increased demand in data transported over networks which peak demands such as streaming of video at home in the evenings and implications on energy requirements for infrastructure. As stated in [13], research that investigates the balance of carbon savings and energy needs in digitization has organized such work into the study of different types of effects:

(i) first-order effects that consider energy needed for producing and using ICT, for example, the energy cost of the consensus mechanism used in a blockchain;
(ii) second-order effects that result from other forms of changes, which may also be influenced by innovations in ICT and its use, for example, changes in travel; and
(iii) tertiary effects that concern the longer-term use of ICT, for example, how regulations, design principles, and deployment measurements can aid sustainability of ICT.

We, therefore, suggest that the sustainability aspects of BCT/DLT are best studied and developed within a larger framework that manages, evaluates and ideally certifies the sustainability of ICT systems in relation to all of the above effect types. Blockchain projects such as SolarCoin [14], which rewards the generation of solar power, could then be evaluated in a methodical way to fully understand their potential contributions to a more sustainable world.

## 3. Papers in this special issue

For sake of illustration, let us now sketch the contributions of some of the papers in this special issue. The paper by McGinn *et al.* [15] argues and demonstrates that the combination of data analytics and data visualization offers a powerful tool box for understanding behaviours and trends on open, permissionless blockchains—here illustrated on Bitcoin.

Cryptocurrencies may be grouped into those whose transactions are based on accounts and those where a transaction redistributes assets from so-called unspent transaction outputs (UTXO). Although these approaches are mathematically equivalent, they differ in behaviour, for example, in terms of information retrieval. The paper by Péres-Solà *et al.* [16] studies some of the most popular UTXO-based cryptocurrencies and identifies scope for improvement in the implementation of the UTXO technology. The aforementioned need for more scalability for transaction processing is the subject of the paper by Burchert *et al.* in [17]. They develop a layer that sits between the blockchain and the payment channel so that channel-based micropayments can be realized with considerably cheaper transaction costs. The resiliency of blockchains to quantum attacks is the subject of the paper by Stewart *et al.* in [18]; in particular, this paper develops an approach for how to securely move funds from a blockchain to a quantum-resistant one even as a quantum attack is taking place on the former. It has been argued by many crypto advocates that cryptocurrencies may serve as a viable alternative to fiat currencies. In the paper [19], Lipton *et al.* develop a framework for such a digital coin that is asset-backed, and so has the means of controlling the stability of this coin through financial mechanisms.

## Data accessibility

Not applicable for this preface.
